# Consumptive hypothyroidism in a patient with malignant rhabdoid tumor of the kidney: case report on a newly found association

**DOI:** 10.1530/ETJ-22-0006

**Published:** 2022-09-27

**Authors:** Roberto Fiore, Stefano La Rosa, Silvia Uccella, Deborah Marchiori, Peter A Kopp

**Affiliations:** 1 Division of Endocrinology, Diabetes and Metabolism, University Hospital of Lausanne and University of Lausanne, Hôtel des Patients, Lausanne, Switzerland; 2 Unit of Pathology, Department of Medicine and Surgery, University of Insubria, Varese, Italy

**Keywords:** hypothyroidism, deiodinase type 3, thyroid hormone, rhabdoid kidney tumor

## Abstract

**Introduction:**

Consumptive hypothyroidism is a rare paraneoplastic condition most commonly associated with infantile hemangiomas. It is caused by overexpression of deiodinase type 3 (D3), which leads to preferential conversion of thyroxine to the metabolically inactive reverse triiodothyronine (rT3), paralleled by a decrease of the biologically active T3.

**Case presentation:**

A 46-year-old male patient with previously normal thyroid function was diagnosed with a renal carcinoma with rhabdoid differentiation. He was treated with sunitinib, followed by the immune checkpoint inhibitors ipilimumab and nivolumab, and he developed primary hypothyroidism secondary to thyroiditis. Substitution with unusually high doses of levothyroxine as high as 4.3 µg/kg/day did not normalize his thyroid function. Poor compliance was refuted because there was no improvement after observed administration. He had no malabsorption. Although tyrosine kinase inhibitors can increase the expression of D3, this effect tends to be modest. Therefore, the suspicion of tumor-related consumptive hypothyroidism was raised and supported by low free T3 and elevated rT3 levels. The therapy could not be further modified because the patient opted for palliative care and passed away 12 days later.

Immunohistochemistry of the tumor from a sample obtained prior to systemic therapy documented abundant expression of D3, corroborating the diagnosis of consumptive hypothyroidism.

**Conclusions:**

This observation extends the spectrum of malignancies overexpressing D3. Although rare, increased awareness of this paraneoplastic syndrome is key, if persistent hypothyroidism cannot be explained by compliance issues or malabsorption. Substitution with high doses of levothyroxine, and combination therapy with liothyronine, can correct hypothyroidism in these patients.

**Established facts**

- Consumptive hypothyroidism is a rare paraneoplastic syndrome, resulting in the degradation of T4 and T3 by overexpression of deiodinase 3 in neoplastic tissue. It has been primarily observed in patients with hemangiomas. It should be considered in subjects with high thyroid hormone requirements after excluding poor compliance and malabsorption.
- Very high doses of levothyroxine, sometimes in combination with liothyronine, are needed to compensate for the increased deiodinase 3 activity.

**Novel insights**

- The observation of consumptive hypothyroidism in a patient with a renal carcinoma with rhabdoid differentiation expands the spectrum of tumors associated with this unusual paraneoplastic manifestation and reports this phenomenon for the first time in an epithelial tumor.

## Introduction

The iodothyronine deiodinases (D1–3) are selenoenzymes that catalyze mono-deiodination on either the outer ring (D1/D2) or the inner ring (D3) of thyroxine (T4). Deiodination of the outer ring generates the active thyroid hormone triiodothyronine (T3), whereas that of the inner ring converts T4 to reverse T3 (rT3), and T3 to 3,3’-diiodo-l-thyronine (T2), which are both biologically inactive.

Overexpression of D3 can lead to increased inactivation of T4 and T3 that exceeds the maximal rate of thyroid hormone synthesis, thereby resulting in so-called *consumptive hypothyroidism* (1). In patients on thyroid hormone substitution, this translates into a requirement for substitution with very high doses of levothyroxine to overcome the massive inactivation of T4, or, alternatively, the need for substitution with liothyronine (LT3). Consumptive hypothyroidism has been mainly reported in highly vascularized hemangiomas in children (2). In adults, a few patients with gastrointestinal stromal tumors overexpressing D3 have been reported (3, 4, 5). It is the first time this rare paraneoplastic condition is reported in a tumor of epithelial origin.

## Case presentation

A 46-year-old man had been diagnosed with a clear cell carcinoma with rhabdoid differentiation (World Health Organization grade 4) of the left kidney in May 2018 through a renal biopsy. The tumor was locally invasive, associated with metastases to retroperitoneal lymph nodes, the liver and the lungs. Because of the widely metastatic disease, nephrectomy was not performed. At initial diagnosis, the thyroid function tests showed a discrete elevation of his thyroid-stimulating hormone (TSH) at 4.5 mU/L (reference range 0.27-4.2 mU/L), with normal levels of fT4 (16 pmol/L, reference range 12.0–22.0 pmol/L) and fT3 (4 pmol/L, reference range 3.1–6.8 pmol/L) (Table 1). He was subsequently started on systemic therapy with the tyrosine kinase inhibitor sunitinib in May 2018, pending approval for immunotherapy with nivolumab and ipilimumab. Therapy with nivolumab was initiated in June, and ipilimumab was added in August. The therapy with sunitinib was definitely stopped in October after completion of the mutational analysis of the primary tumor (next-generation 400 gene sequencing panel), which showed mutations in the *TERT* promoter and the *MET* gene (exon 15: p.V1088A), and amplification of *SETD2* (SET domain containing 2, histone-lysine N-methyltransferase) and *MET*. Moreover, the tumor showed significant expression of programmed death ligand 1 (80%).

**Table 1:** Synopsis of the thyroid function tests in a patient with consumptive hypothyroidism associated with a malignant rhabdoid tumor of the kidney.

Date	Oncologic treatment	Thyroid replacement	TSH mUI/L (0.27–4.2)	Free T3 pmol/L (3.1–6.8)	Free T4 pmol/L (12–22)	Total T3 nmol/L (1.3–3.1)	Total T4 nmol/L (66–181)	Reverse T3 nmol/L (0.14–0.54)
15.05.2018			4.5	4.0	16.0			
28.05.2018	Start sunitinib							
25.06.2018	Start nivolumab							
23.07.2018			4.69					
16.08.2018	Start ipilimumab							
27.08.2018			0.025					
29.08.2018				7.3	39		184	
18.09.2018			0.006	7.1	42			
16.10.2018	Stop sunitinib							
30.10.2018			26.3	2	9			
09.11.2018	Stop ipilimumab	Start levothyroxine 100 µg/day						
07.12.2018	Start axitinib		72.7					
04.01.2019			81.5	1.5	9			
22.01.2019			66.6					
24.01.2019	Stop axitinib							
12.02.2019			74.8					
18.02.2019		Increase to 300 µg/day						
28.02.2019	Last cycle nivolumab							
05.03.2019			80.5	1.1	10.4			
15.03.2019			85	1.1	8.2	0.5	46	0.79

Shortly after the introduction of ipilimumab, he developed transient thyrotoxicosis secondary to immune checkpoint inhibitor-associated thyroiditis, with subsequent hypothyroidism (October 2018: TSH 26.3 mU/L, fT4 9 pmol/L, fT3 2.0 pmol/L). At this stage, levothyroxine substitution was introduced (100 µg/day, corresponding to about 1.3 µg/kg/day). However, the TSH levels further increased to 72.7 mU/L after 4 weeks of therapy. The situation remained unchanged in the following months; in January 2019, the TSH was 81.5 mU/L, the fT4 was decreased at 9 pmol/L, and the fT3 was particularly low at 1.5 pmol/L. On February 18, 2019, the levothyroxine dosage was increased to 300 µg/day (corresponding to 4.3 µg/kg/day). Despite that, the fT4 and fT3 levels remained at similar levels after 2 weeks with 10.4 and 1.1 pmol/L.

The patient was hospitalized in March 2019 with a diffuse atopic eczema and was found to be severely anemic. He was hypotensive and tachycardic, and the dermatologic exam revealed dry skin and eczema eruptions with erythematous and squamous papules and plaques.

At that time, endocrinology was consulted for the first time. The patient reported profound weakness, a weight loss of 14 kg during the last 8 months, constipation, and a generalized pruritic rash. He plausibly reported regular and appropriate intake of the levothyroxine. Of note, the patient did not present any symptoms of malabsorption. At that time, the patient intermittently took proton pump inhibitors but took no other medication known to interfere with thyroid hormone absorption or metabolism.

Although he then took the levothyroxine under supervision during a week, the thyroid function tests did not improve: fT4 was 8.2 pmol/L, fT3 1.1 pmol/L, total T4 46 nmol/L (reference range 66–181 nmol/L), and total T3 0.5 nmol/L (reference range 1.3–3.1 nmol/L). Suspecting that the patient might have consumptive hypothyroidism, rT3 was ordered and immunohistochemistry of the tumor for D3 expression was planned. The rT3 level drawn 9 days prior to his passing but only available about a month later was found to be elevated at 0.79 nmol/L (reference range 0.14–0.54 nmol/L). Of note, the estimated increase of his tumor burden based on cross-sectional imaging studies was about 57% between June 2018 and March 2019 (557.01 cm^3^ to 878.73 cm^3^ based on RECIST (Response Evaluation Criteria in Solid Tumors) criteria).

The patient and his family opted for palliative care and he left the hospital; he passed away shortly thereafter at home.

## Immunohistochemistry

Histopathological investigation of the primary tumor was completed with an immunohistochemical analysis, performed on formalin-fixed paraffin embedded tissue in an automated stainer (Benchmark XT, Ventana Medical Systems, Tucson, AZ, USA) using the following antibodies: CD10 (clone 56C6, Novocastra, New Castle, UK), cytokeratin (CK) 7 (clone OV-TL 12/30, Dako), panCK (clone AE1/AE3, Dako), PAX8 (polyclonal, Proteintech, Manchester, UK), vimentin (clone VIM3B4, PROGEN Biotechnik, Heidelberg, Germany), and alpha-methylacyl CoA racemase protein p504s (clone 13H4, Biologo, Kronshagen, Germany). In order to verify the D3 expression, immunohistochemistry with a specific affinity-purified polyclonal rabbit antibody ( catalog no. NB110-96414, Novus Biologicals, Centennial, CO, USA) was performed, as previously described (6).

## Results

**Figure 1:**
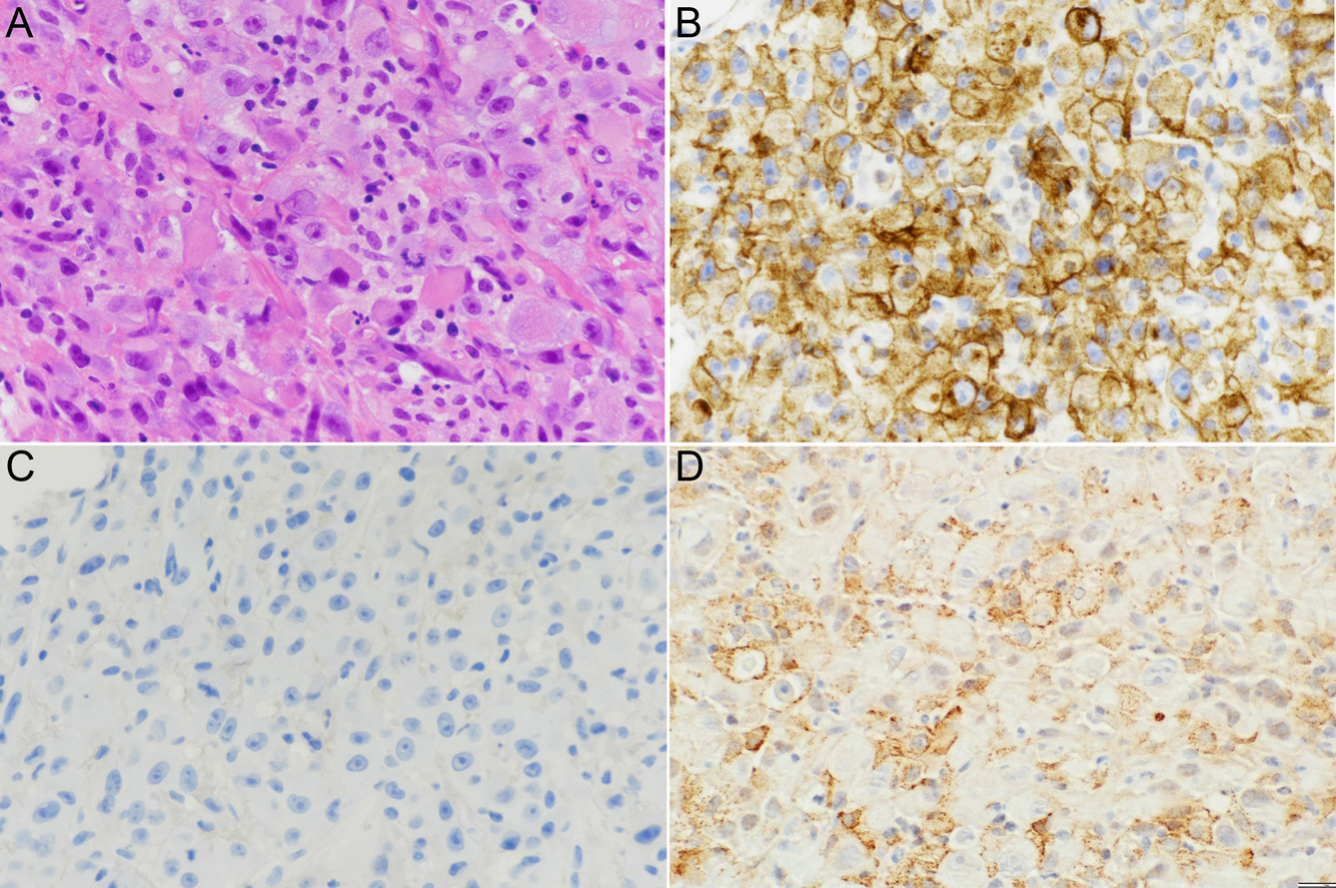
Histopathological aspects of the renal carcinoma with rhabdoid features. (A) Hematoxylin and eosin stain; the histology shows a solid proliferation of highly atypical cells, a subset of which are of large size, polygonal, with eccentric round nuclei and abundant eosinophilic cytoplasm with a paranuclear globular structure, conferring the typical rhabdoid aspect. (B) Intense immunoreactivity for CD10 and (C) lack of immunostaining for cytokeratin 7 are in support of the diagnosis. (D) Immunohistochemical staining for D3 is present in numerous neoplastic cells as granular brown paramembranous cytoplasmic precipitate (hematoxylin and eosin and immunoperoxidase, x200).

Histologically, the tumor was characterized by a solid proliferation of highly atypical cells with clear to eosinophilic cytoplasm and pleomorphic nuclei with prominent nucleoli. A subset of cells were large and polygonal with abundant eosinophilic cytoplasm and a paranuclear globular structure and eccentric round nuclei, conferring the typical rhabdoid aspect (Fig. 1A). Tumor cells were positive for CD10 (Fig. 1B), PAX8, vimentin, and panCK, while they were negative for CK7 (Fig. 1C) and p504s.

Immunoreactivity for D3 was visible as moderately intense granular cytoplasmic brown stain in about 40% of neoplastic cells (Fig. 1D). Stromal cells, including inflammatory cells, endothelia, and fibroblasts, were negative.

## Discussion

The patient presented here developed severe hypothyroidism resistant to high-dose substitution of levothyroxine. While thyroiditis secondary to systemic therapy initiated the overt thyroid dysfunction, a frequently observed phenomenon (7, 8), the unusually high thyroid hormone requirements, the abundant expression of D3 in the tumor present already prior to therapy, and the elevated rT3 levels are consistent with concomitant consumptive hypothyroidism.

At initial presentation, prior to any therapy, the patient only had a slightly elevated TSH and normal peripheral hormones. This constellation suggests that the thyroid was able to compensate for an increased degradation of T4 and T3 at that time. The subsequent loss of his thyroid function secondary to immunotherapy-related thyroiditis, coupled with progression of the disease (increase of the tumor burden of 57%, from 557.01 cm^3^ to 878.73 cm^3^ based on RECIST criteria), and, hence, an increase in overall D3 activity, then led to the constellation characteristic for consumptive hypothyroidism.

The observation of consumptive hypothyroidism associated with a renal carcinoma with rhabdoid differentiation, for the first time a neoplasia of epithelial origin, further expands the spectrum of tumors associated with this unusual paraneoplastic manifestation.

Consumptive hypothyroidism was first described by Huang *et al.* in a 3-month-old infant with multiple liver hemangiomas with overexpression of D3 (9). A review from 2017 identified 42 cases of consumptive hypothyroidism; 36 of them were found in children (2). The vast majority (97%) of pediatric patients had vascular tumors such as hemangiomas and hemangioendotheliomas. In contrast, among the six cases in adults, four had solid nonvascular tumors (gastrointestinal stromal tumor (GIST) (*n*  = 2) or fibrous tumors (*n*  = 2)) (3, 5, 10, 11). Since 2017, this paraneoplastic condition has been reported in another 11 pediatric patients with hemangiomas, but only two additional adults, one with a GIST (4) and one with a hemangiopericytoma (12). In addition to the high thyroid hormone requirements with characteristic thyroid function tests, the diagnosis can be confirmed by demonstrating overexpression of D3.

D3 is one of the three human iodothyronine selenodeiodinases and has been cloned in 1994 (13). It has mainly inner ring deiodination (IRD) activity which is fundamental for thyroid hormone catabolism, while D1 (which also has weak IRD capacity) and D2 have outer ring deiodination activity, which is essential for thyroid hormone activation from T4 to T3 and catabolism of rT3 to T2. D3 is physiologically expressed in the brain, liver (only in the fetus), ovary, skin, testes, uterus, and the placenta (14). In the placenta, D3 activity decreases gradually during pregnancy, although total expression increases, in order to regulate maternal transfer of thyroid hormone to the fetus.

The overexpression of D3 in tumor tissue can lead to hypothyroidism whenever its activity exceeds the maximal synthesis capacity of the thyroid. Such patients require unusually high doses of thyroid hormone substitution (an average dose of 300 µg/day, with or without LT3) to achieve euthyroidism (2). This is also illustrated by the patient presented here, in whom primary hypothyroidism was first induced by systemic therapy with checkpoint inhibitors, and then further complicated by consumptive hypothyroidism. In this context, it is worthwhile mentioning that D3 activity can be increased by therapy with tyrosine kinase inhibitors (7, 15, 16). For example, Abdulrahman *et al.* reported that the required levothyroxine dose increased from 2.48 µg/kg/day to 2.71 µg/kg/day after treatment with sorafenib for 26 weeks in 21 patients (7). However, this increase in levothyroxine is modest compared to the patient reported here whose TSH did not normalize with a dose as high as 4.3 µg/kg/day.

Infants and children with consumptive hypothyroidism pose a particular therapeutic challenge since insufficient thyroid hormone substitution can impact neurocognitive development and can be responsible for a decrease in the intelligence quotient (9).

Although consumptive hypothyroidism is overall a very rare paraneoplastic syndrome, particularly in adults, endocrinologist and oncologists should be aware of this condition which can be associated with vascular tumors, but also solid tumors such as GIST, fibrous tumors, and, as presented here, malignant rhabdoid kidney tumors.

## Declaration of interest

The authors declare that there is no conflict of interest that could be perceived as prejudicing the impartiality of this case report.

## Funding

This work did not receive any specific grant from any funding agency in the public, commercial, or not-for-profit sector.

## Statement of ethics

The patient had signed the general informed consent to participate in research upon admission to the University Hospital of Lausanne.

## Author contribution statement

All persons listed as authors made significant contribution to the writing and critical revision of this case report and approved the final version for submission for publication.
